# Impact of lymph nodal stage on gallbladder cancer survival after extended cholecystectomy and adjuvant radiochemotherapy: long-term results from an oncology institute, Chile

**DOI:** 10.3332/ecancer.2021.1222

**Published:** 2021-04-22

**Authors:** Manuel González-Domingo, Benjamín Omegna Lafuente, Álvaro Rojas Luca, Pablo Kirmayr Zamorano, Pablo González Mella

**Affiliations:** 1 Radiotherapy Department, Instituto Oncológico, Hospital Naval Almirante Nef, Clínica Reñaca, Anabaena 336, Jardín del Mar, Reñaca, Viña del Mar, Valparaíso, Chile; 2 Universidad de Valparaíso, Hospital Naval Almirante Nef, Viña del Mar 2520000, Chile; 3 Digestive Surgery Service, Hospital Naval Almirante Nef, Viña del Mar 2520000, Chile; 4 Radiotherapy Department, Instituto Oncológico FALP, Santiago 7500691, Chile

**Keywords:** gallbladder cancer, radio-chemotherapy, surgery, lymph nodes

## Abstract

**Introduction:**

Gallbladder cancer (GBC) is one of the most important causes of cancer death in Chile.

**Materials and methods:**

A retrospective review of 103 patients with a diagnosis of GBC who were treated with surgery and adjuvant radiochemotherapy (RT-CT) was carried out at the Oncological Institute of Viña del Mar, Chile. Of these, 56 underwent surgery with oncological criteria, in which the impact of lymph node involvement and prognostic factors for survival were analysed.

**Results:**

The median follow-up was 47.5 months. The 5-year survival of the patients operated on with oncological surgery was 55%, and for those resected without oncological criteria, it was 32% (*p* = 0.02). Regarding the impact of lymph node involvement, 5-year overall survival (OS) in patients with compromised lymph nodes was 32% versus 68% for patients without compromised lymph nodes (*p* = 0.006). Five-year OS in patients without involved nodes, with 1 involved node or with>1 involved node was 68%, 44% and 12%, respectively (*p* = 0.0002). The N ratio was grouped in 0, <10% and ≥10%. Five-year OS was 71%, 0% and 24%, respectively (*p* = 0.003). There was no evidence of differences in survival with respect to the number of lymph nodes studied.

**Conclusion:**

Our data provide information regarding the importance of lymph node involvement in patients with GBC undergoing surgery with oncological criteria and adjuvant RT-CT. In the absence of randomised studies, it is suggested to have a more aggressive therapeutic approach in those patients with two or more involved nodes or with a lymph node ratio >10%.

## Introduction

Gallbladder cancer (GBC) is a rare neoplastic disease worldwide. In 2018, 219,420 new cases and 165,087 deaths were estimated worldwide [1].

Along with other countries such as India and Japan, Chile is considered an area with a high incidence of this pathology, being the major cause of cancer-related mortality in Chilean women, with a rate of 16.1/100,000 [2].

In its early stages, GBC is an aggressive and asymptomatic pathology. Data from the National Cancer Database reports 5-year survival of 60% in early stages, and 5% in patients with serosa or lymph node involvement [3].

The only curative treatment is surgery, which is limited to 10%–25% of patients [4], due to the presence of tumour involvement of the liver pedicle, infiltration of locoregional organs or the presence of distant lymph nodes, which contraindicate curative surgery in most cases.

Due to its low incidence worldwide, there were no standardised guidelines until 2015, when the International Hepato-Pancreato-Biliary Association published its expert consensus [5].

This consensus establishes that standard lymphadenectomy should include the lymph nodes of the hepatoduodenal ligament and the periportal, recommending a harvest of at least six lymph nodes, in order to determine the correct stage of the disease.

The objective of this review is to analyse prognosis factors for survival, with emphasis on lymph node stage, in patients with GBC who had undergone extended cholecystectomy and who had received adjuvant radiochemotherapy (RT-CT).

## Materials and methods

### Patients

A retrospective review of the patients with a diagnosis of GBC who were treated with oncological surgery and adjuvant RT-CT was carried out at the Oncological Institute of Viña del Mar, Chile. It included patients with tumours infiltrating the muscle or deeper layers and/or compromised nodes, with no evidence of distant metastasis.

### Treatment

The extended cholecystectomy was performed via laparotomy, and consisted on an IVb-V hepatic bisegmentectomy or wedge resection of the tumour bed, plus standard lymphadenectomy, which includes lymph nodes of the cystic duct, common hepatic duct, periportal and hepatic artery. The surgery began with sampling of the aortocaval nodes and only if these were free of tumour in the intraoperative biopsy, the aforementioned procedure was continued. The section border of the cystic duct was also sent for frozen section. In case of a positive result, resection of the distal bile duct was carried out.

### Lymph node status evaluation

Three parameters were used to assess node status: total lymph node count, number of positive lymph nodes and the proportion of positive lymph nodes versus total lymph nodes harvested (lymph node ratio (LNR)).

### Adjuvant treatment

All patients were treated with 3-dimensional conformational radiotherapy with high-energy dual linear accelerator (6–10 MV), and treated at 1.8 Gy per fraction, 5 days a week for up to 40–50 Gy to the tumour bed and regional nodes. In the first years of treatment, some patients received the total abdominal irradiation technique (whole abdominal irradiation (WAI)). Chemotherapy was based on fluoropyrimidines, mainly oral capecitabine.

Toxicity was measured according to the Common Criteria for Toxicity of the National Cancer Institute.

Discharged patients were regularly followed up in outpatient clinics, for 1–6 months for at least 5 years, with a median follow-up of 47.5 (range 2–233) months.

### Statistic analysis

Survival was calculated using the Kaplan-Meier method: a log-rank test was used to calculate differences using 5% or less as a significant value. Multivariate analysis was performed using the Cox proportional hazards model. Calculations were performed with the STATA statistical software, version 16.

## Results

Between December 1991 and July 2019, 103 patients with a diagnosis of GBC were treated with surgery and adjuvant RT-CT at the Oncological Institute of Viña del Mar, Chile. Out of these, 56 (54%) patients underwent surgery with oncological criteria (extended cholecystectomy). In 5 (9%) it was performed in the first intervention (pre or intraoperative suspicion), while 51 (91%) patients were re-operated (incidental diagnosis).

**Figure 1.**
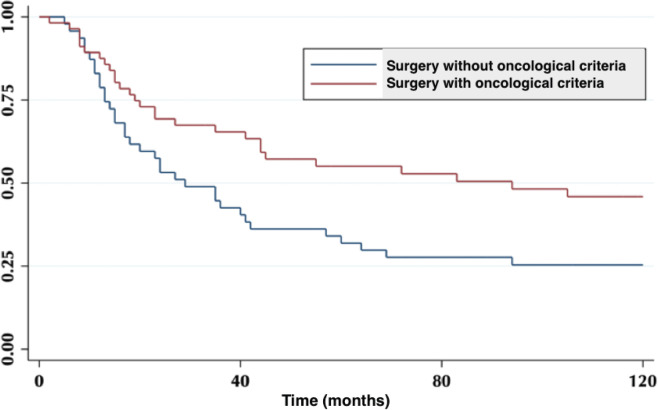
Five-year survival in patient with oncological surgery and without oncological surgery.

Five-year survival for patients resected without oncological criteria was 32%. For those resected with oncological surgery, it was 55% (*p* = 0.02) (Figure 1). Of the latter, the overall survival (OS) at 2, 3 and 5 years was 83.5%, 62% and 44%, respectively.

Regarding the group treated with oncological surgery, 39 were women (70%), with an average age of 59 years (range 32–76 years). Sixteen (29%) presented compromise of the muscular layer (T1b), 22 (39%) compromise of the subserosa (T2) and 18 (32%) the tumour reached the serosa (T3). Nodal involvement was found in 21 cases (43%). The rest of the demographic characteristics of the patients are described in Table 1.

**Figure 2.**
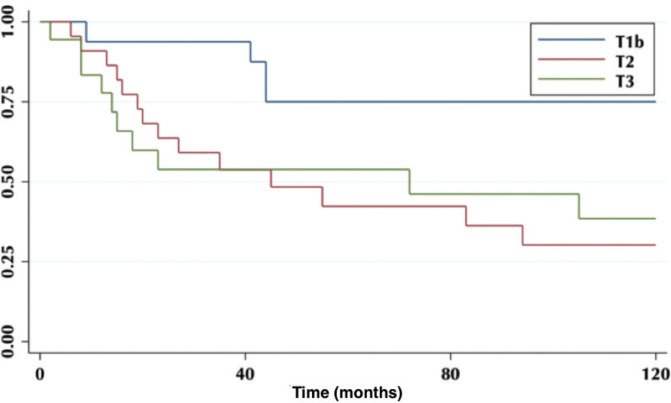
Five-year survival according to tumour infiltration.

According to the degree of tumour infiltration, the overall 5-year survival for T1b patients was 75%, T2 42% and for T3 54% (*p* = 0.04) (Figure 2).

**Figure 3.**
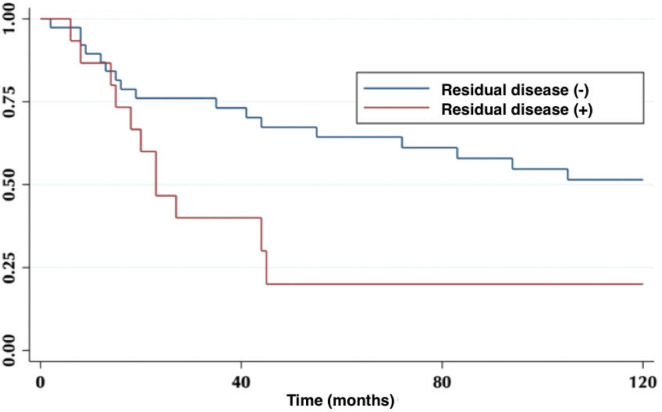
Patient 5-year survival with and without residual disease.

The 5-year OS in patients with or without residual disease in the liver bed was 20% versus 64%, respectively (*p* = 0.01) (Figure 3).

Regarding the prognosis according to lymph node status, it was evidenced that the 5-year OS in patients without studied nodes was 51% versus 56% in patients with one or more studied nodes (*p* = 0.63).

Considering the group of patients with harvested nodes, the 5-year OS in patients with <3 studied nodes was 48% versus 59% for patients with three or more studied nodes (*p* = 0.84). In patients with <6 studied nodes, it was 51% versus 77% for patients with six or more studied nodes (*p* = 0.82).

**Figure 4.**
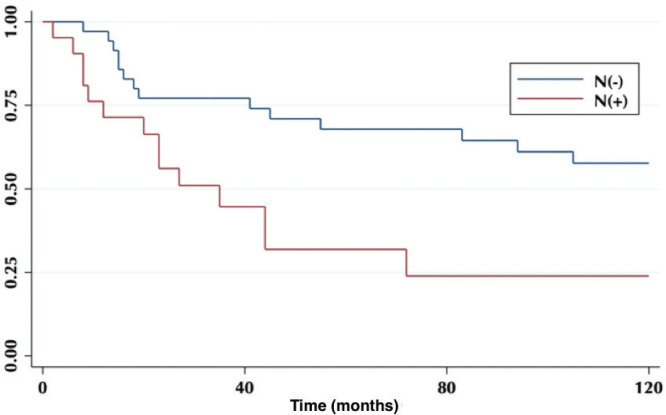
Five-year survival in a patient with and without compromised lymph nodes.

**Figure 5.**
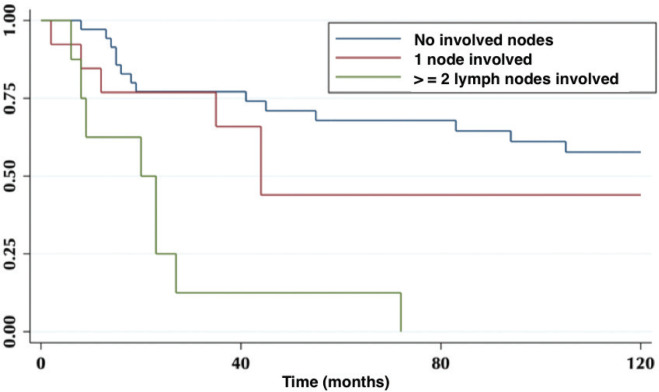
Five-year survival in patients without involved nodes, with one involved node and with two or more involved nodes.

According to lymph node positivity, the 5-year OS in patients with compromised lymph nodes was 32% versus 68% for patients without compromised lymph nodes (*p* = 0.006) (Figure 4). Comparison according to the number of involved nodes shows that 5-year survival in patients with one involved node versus patients with more than one involved node was 44% and 12%, respectively (*p* = 0.0002) (Figure 5).

**Figure 6.**
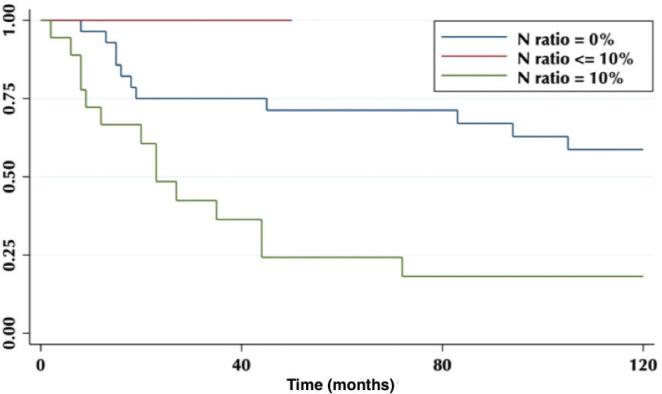
Five-year survival according to the proportion of lymph nodes involved.

The lymph node N ratio was grouped in 0, <10% and ≥10%. The 5-year OS was 71%, 0% and 24%, respectively (*p* = 0.003) (Figure 6).

Regarding the time elapsed between the first surgery and the subsequent surgery with oncological criteria, the 5-year survival of patients reoperated in less than 6 weeks versus those reoperated in more than 6 weeks was 51% and 62%, respectively (*p* = 0.35).

The univariate analysis of survival predictors in patients undergoing surgery with oncological criteria is summarised in Table 2.

## Discussion

In Chile, we have a high incidence of cholelithiasis, which is diagnosed with abdominal ultrasound, with almost the majority of diagnoses of GBC [6] are incidental (50%–75%), rather than suspicion of cancer. Our review included patients who were diagnosed from 1993 to date, so the advancement in images has been important in recent years, possibly that may be a factor why they were not diagnosed before the operation, since currently ultrasound is sensitive enough to detect these tumours.

The high percentage of laparotomies as the first surgery is due to the fact that laparoscopy in Chile began to become widespread in the last decades. This is why a large part of the patients operated at the beginning of our work were by laparotomy, with the progressive increase of the laparoscopic route.

The demographic characteristics of our sample were very similar to that described by international publications [7].

The incidence of residual disease varies according to the T classification of the primary tumour [8]. It is lower, in ranges from 0% to 12%, in patients with T1 tumours; reaching up to 46% in those with T3 tumours. Regarding re-operated patients, 27% had liver bed compromise, and their OS was statistically lower compared to those without residual disease (Figure 3).

A review of the risk of lymph node involvement, distant metastasis and OS figures was performed in this group of patients (T1b). The risk of lymph node involvement is close to 15%, and 50 of the patients present histological elements that could confer a high risk (lymphatic and/or perineural vascular permeation). The 5-year mortality figures, even after radical surgery, are close to 15% [8–11].

Which should be the standard of surgeries with oncological criteria for these patients is still a matter of controversy. Given the absence of randomised controlled trials, and the low level of evidence obtained in systematic reviews, the definition of radical resection in most guidelines for GBC varies in different countries. With the consensus published in 2015, the management has been universally standardised [5].

The year we started adjuvant treatment in patients with GBC was 1993, at that time it was not standard to perform wedge resection of the liver segment or lymphadenectomy, for this reason, despite being suggested to surgeons in the area, it was not done in a standard way, but if we did adjuvant RT-CT knowing that the results were bad with that surgery alone.

The goal of liver resection is to achieve R0 resection. The routine performance of major hepatectomy compared with partial hepatectomy (non-anatomical resection of the gallbladder bed) or a formal segmentectomy (segment IVB-V resection) has not been associated with survival benefits, but with an increase in morbidity [12].

Lymph node involvement (nodal status) is an established prognostic factor in several gastrointestinal tumours [13]. There are three conventional parameters that describe nodal status: the anatomical location of the involved nodes, the number of involved nodes and the lymph node ratio or LNR. Which of the three parameters of lymph node status best stratifies patients with GBC according to prognosis remains controversial [14].

The experts consensus published in 2015 recommends a harvest of at least six lymph nodes for adequate staging. This recommendation is based on the study published by Ito *et al* [16] in which 120 patients with GBC were analysed with a median follow-up of 23 months, of which 41 (34%) had compromised lymph nodes, with an average of three lymph nodes harvested [15]. The low harvest of lymph nodes is repeated in most of the studies published in literature, with only 5% lymphadenectomy of three or more nodes [13, 16].

In our review, lymph node harvest was inadequate in most cases, similar than in the most of the surgical series published in the literature [13, 17]. There are no randomised studies addressing the issue of surgery and we have only retrospective analysis and a recently published consensus [5].

In our review, the number of lymph nodes studied was not a survival predictor. No differences in survival were found when studying one or more nodes, more than two nodes or more than five nodes.

On the other hand, lymph node involvement was a predictor of survival (Figure 4). When comparing the number of involved nodes, it is evidenced that having two or more involved nodes, 5-year survival drops drastically to 12%, which would lead to suggest that in these patients a more aggressive approach to treatment should be taken, with the study of genetic or molecular factors that allow targeted therapies (immunotherapies, target therapies) to improve their ominous prognosis.

Regarding the LNR, our results show that an LNR ≥10% is a factor of poor prognosis (Figure 6), which is correlated with other published studies [14], and due to the constant low lymph node harvest, it becomes a useful tool to determine therapeutic behaviours.

The type of adjuvant treatment is still controversial given the lack of randomised studies. A systematic review of the effectiveness of adjuvant therapies in patients with GBC shows that in patients with lymph nodes and/or compromised surgical margin, adjuvant treatment would provide benefit in OS [18].

Different studies and reviews show that adjuvant radiotherapy improves the prognosis of patients with locally advanced GBC [19–22] and it is the therapy with the highest published clinical evidence, together with concomitant chemotherapy, in our environment [23–25].

Data shown here reveals the impact of the lymph node stage on the prognosis of patients with GBC treated with oncological surgery and adjuvant RT-CT. On the one hand, they suggest the need to try a more specific and sensitive pre-operative study to be able to detect lymph node involvement prior to oncological surgery. On the other hand, given the poor survival prognosis, they suggest to propose some neoadjuvant treatment, with the approach of achieving pathological response of the lymph node and liver bed, and thus evaluating its impact on survival in this context.

## Conclusion

Our data provides relevant information regarding the importance of lymph node involvement in patients with GBC undergoing surgery with oncological criteria and adjuvant RT-CT. In the absence of randomised studies, this data suggests having a more aggressive therapeutic approach in those patients with two or more involved nodes or with an LNR>10%. On the other hand, there is an urgent need for a more exhaustive pre-operative study, in order to detect patients with lymph node involvement and thus being able to offer them possible neoadjuvant therapy.

## Funding statement

The review has been self-funded.

## Conflicts of interest

No conflicts of interest exist.

## Figures and Tables

**Table 1.** Demographic characteristics of patients undergoing surgery with oncological criteria.

Parameter	Variable	*n* (100%)
Sex	Female	39 (70%)
	Male	17 (30%)
Age (years)	Median (range)	59 (32–76)
First surgery	Elective	41 (73%)
	Emergency	15 (27%)
First surgery technique	Laparoscopy	31 (55%)
	Laparotomy	25 (45%)
Tumour	Ib	16 (29%)
	II	22 (39%)
	III	18 (32%)
Histological grade	GI	25 (45%)
	GII	18 (32%)
	GIII	13 (23%)
Time between surgeries (weeks)	<6	33 (59%)
	>6	23 (41%)
Liver resection type	Wedge	52 (93%)
	Bisegmentectomy	3 (5.5%)
	Major surgery	1 (1.5%)
Residual disease	No	41 (73%)
	Yes	15 (27%)
Number of lymph nodes studied	0	7 (13%)
	1–2	11 (20%)
	3–6	26 (46%)
	>6	12 (21%)
Number of involved nodes	0	28 (57%)
	1	13 (26.5)
	>1	8 (16.5%)
LNR^a^	0	28 (57%)
	<10	12 (24%)
	>10	9 (18%)

a LNR, Lymph node ratio

**Table 2.** Univariate analysis of predictive survival factors in patients undergoing surgery with oncological criteria.

Parameter	Variable	OS^a^ 5 years	Statistical significance
Stage T	IB	75%	*p* = 0.04
	II	42%	
	III	54%	
Time between surgeries (weeks)	<6	51%	*p* = 0.35
	>6	62%	
Residual disease	No	64%	*p* = 0.01
	Yes	20%	
Number of lymph nodes studied	0	51%	*p* = not significant
	³1	56%	
	³3	59%	
	³6	77%	
Nodal involvement	N0 N+	68% 32%	*p* = 0.006
Number of involved nodes	0	68%	*p* = 0.002
	1	44%	
	³2	12%	
LNR^b^	0	71%	*p* = 0.003
	<10	0%	
	³10	24%	

a OS, Overall survival

b LNR, Lymph node ratio
